# Effect of implementing care protocols on acute ischemic stroke outcomes: a systematic review

**DOI:** 10.1055/s-0042-1759578

**Published:** 2023-03-22

**Authors:** Karina Fonseca de Souza Leite, Mariana Gaspar Botelho Funari de Faria, Rubia Laine de Paula Andrade, Keila Diane Lima de Sousa, Samuel Ribeiro dos Santos, Kamila Santos Ferreira, Carlos Eduardo Menezes de Rezende, Octavio Marques Pontes Neto, Aline Aparecida Monroe

**Affiliations:** 1 Universidade de São Paulo, Escola de Enfermagem de Ribeirão Preto, Departamento de Enfermagem Materno-Infantil e Saúde Pública, Ribeirão Preto SP, Brazil.; 2 Universidade de São Paulo, Faculdade de Medicina de Ribeirão Preto, Hospital das Clínicas, Ribeirão Preto SP, Brazil.; 3 Ministério da Saúde, Agência Nacional de Saúde Suplementar, Brasília DF, Brazil.; 4 Universidade de São Paulo, Faculdade de Medicina de Ribeirao Preto, Departamento de Neurociências e Ciências do Comportamento, Ribeirão Preto SP, Brazil.

**Keywords:** Ischemic Stroke, Acute Disease, Emergencies, Clinical Protocols, Treatment Outcome, AVC Isquêmico, Doença Aguda, Emergências, Protocolos Clínicos, Resultado do Tratamento

## Abstract

**Background** Implementing stroke care protocols has intended to provide better care quality, favor early functional recovery, and achieving long-term results for the rehabilitation of the patient.

**Objective** To analyze the effect of implementing care protocols on the outcomes of acute ischemic stroke.

**Methods** Primary studies published from 2011 to 2020 and which met the following criteria were included: population should be people with acute ischemic stroke; studies should present results on the outcomes of using protocols in the therapeutic approach to acute ischemic stroke. The bibliographic search was carried out in June 2020 in 7 databases. The article selection was conducted by two independent reviewers and the results were narratively synthesized.

**Results** A total of 11,226 publications were retrieved in the databases, of which 30 were included in the study. After implementing the protocol, 70.8% of the publications found an increase in the rate of performing reperfusion therapy, such as thrombolysis and thrombectomy; 45.5% identified an improvement in the clinical prognosis of the patient; and 25.0% of the studies identified a decrease in the length of hospital stay. Out of 19 studies that addressed the rate of symptomatic intracranial hemorrhage, 2 (10.5%) identified a decrease. A decrease in mortality was mentioned in 3 (25.0%) articles out of 12 that evaluated this outcome.

**Conclusions** We have identified the importance of implementing protocols in increasing the performance of reperfusion therapies, and a good functional outcome with improved prognosis after discharge. However, there is still a need to invest in reducing post-thrombolysis complications and mortality.

## Resumo

**Antecedentes** A implementação de protocolos de acidente vascular cerebral (AVC) visa proporcionar uma melhor qualidade da assistência, favorecer a recuperação funcional precoce e alcançar resultados para a reabilitação do paciente.

**Objetivo** Analisar o efeito da implantação de protocolos nos desfechos do AVC isquêmico agudo.

**Métodos** Foram incluídos estudos primários publicados entre 2011 e 2020 e que atendiam aos seguintes critérios: população deveria ser constituída de pessoas com AVC isquêmico agudo; apresentar resultados sobre os desfechos do uso de protocolos na abordagem terapêutica ao AVC isquêmico agudo. A busca bibliográfica foi realizada em junho de 2020 em 7 bases de dados. A seleção dos artigos foi feita por dois revisores independentes e a síntese dos resultados foi feita de forma narrativa.

**Resultados** Foram recuperadas 11.226 publicações, das quais 30 foram incluídas no estudo. Após a implementação do protocolo, 70,8% das publicações constataram aumento na taxa de realização de terapia de reperfusão, como a trombólise e a trombectomia; 45,5% identificaram melhora no prognóstico clínico do paciente; e 25,0% dos estudos identificaram diminuição no tempo de internação hospitalar. De 19 estudos que abordaram a taxa de hemorragia intracraniana sintomática, 2 (10,5%) identificaram diminuição nesta taxa. A diminuição da mortalidade foi citada em 3 (25,0%) artigos de 12 que avaliaram tal desfecho.

**Conclusões** Identificou-se a importância da implantação de protocolos no aumento da realização das terapias de reperfusão, e ao bom desfecho funcional com melhora do prognóstico após a alta. No entanto, ainda há que se investir na diminuição das complicações pós trombólise e da mortalidade.

## INTRODUCTION

Stroke is defined as a cerebrovascular disease in which there is a sudden neurological deficit secondary to a brain injury of ischemic or hemorrhagic origin, ranking second among the causes of death worldwide. 1 2 3 The World Health Organization (WHO) defines stroke as a pathology that presents central nervous system dysfunction symptoms that can lead to death or functional sequelae, providing a high chance of disability. 3 A stroke can present itself in two ways: hemorrhagic or ischemic. The latter will be addressed in this study and originates from a blood vessel obstruction causing an interruption of blood flow in a certain brain region and resulting in the loss of its momentary or permanent function 4_._

The recommended therapies for ischemic stroke (I-stroke) are time-dependent and require implementing care protocols that prioritize getting victims to arrive at a medical center in a timely manner and have quick access to a definitive diagnosis. Treatment is based on performing a recanalization procedure, dissolving the thrombus or the occlusive embolus by chemical (systemic or intra-arterial use of thrombolytics) or mechanical thrombolysis (removing clots through a surgical procedure [thrombectomy]). After such procedures, victims must be transferred to a monitored bed, preferably in a Stroke Unit, for continuity of care. 5 6 7

Faced with a short therapeutic window provided by rapid and systematic medical care, the chance of sequelae is proportionally smaller the shorter the time the care is provided to a patient with suspected stroke. 7 Thus, a wide variety of initiatives have facilitated countless efforts in the quality of care provided to these patients, with efforts to provide the shortest time interval between the onset of symptoms and the start of treatment, culminating in a greater chance of a good prognosis. 8 9

In this sense, implementing protocols has been proposed with the intention of enforcing the time goals in relation to the therapeutic window established by The National Institute of Neurological Disorders and Stroke (NINDS) and recommended by the American Heart Association/American Stroke Association (AHA/ASA), 6 10 and consequently provide better care quality and good practices in the care of ischemic stroke patients, favoring early functional recovery and achieving long-term results for the rehabilitation of the patient. 8

Considering this, the present study aims to synthesize and analyze the scientific knowledge produced about the effect of implementing care protocols on the outcomes of acute ischemic stroke.

## METHODS

The present study is a continuation of the study “Reducing care time after implementing protocols for acute ischemic stroke: a systematic review,” accepted for publication in this journal. A systematic review of the literature was conducted according to the Preferred Reporting Items for Systematic Reviews and Meta-Analyses (PRISMA). 11 This type of review is conducted in several stages and has high methodological rigor with a comprehensive and nonbiased approach in order to compile information available in the literature on a specific topic. 12

The PICO strategy, whose acronym was coined by The Joanna Briggs Institute, 13 was used to prepare the following guiding question for the review: What is the effect of implementing care protocols on the outcomes of acute ischemic stroke?; In which: P (population) comprises patients with acute ischemic stroke; I (intervention) is in regard to emergency care protocols; C (comparison) comprises the periods before and after implementing the protocols; and O (outcome) covers case outcomes.

The following inclusion criteria were defined to select the studies: studies in Portuguese, English, and Spanish; articles whose study population consisted of people who had acute ischemic stroke; articles published from 2011 to 2020 and that addressed outcomes of acute ischemic stroke treatment before and after implementing protocols, including: thrombolysis rate, thrombectomy rate, length of hospital stay, case prognosis through the modified Rankin Scale, symptomatic intracranial hemorrhage rate and death rate. Articles not found in full, duplicates, technical productions (manuals, protocols), and descriptive and secondary studies (reviews) were excluded.

The bibliographic search was carried out in June 2020 in the following databases: Excerpta Medica dataBASE (Embase - https://www.embase.com ), Scopus, owned by Elsevier ( https://www.scopus.com ), MEDLINE or Publisher Medlin (accessed through the PubMed platform - https://pubmed.ncbi.nlm.nih.gov/ ) and Latin American and Caribbean Literature in Health Sciences (LILACS - accessed through the Regional Portal of the Virtual Library in Health - https://pesquisa.bvsalud.org/portal/advanced ). Finally, the searches performed in the Cumulative Index to Nursing and Allied Health Literature (CINAHL), Academic Search Premier (ASP) and SocINDEX databases were performed simultaneously through the EBSCOhost platform accessed by the website Periódicos CAPES ( https://www . periodicals.capes.gov.br). This platform automatically deletes the duplicates found in these databases. Vocabularies in Portuguese, English and Spanish were used in the searches carried out in LILACS, while vocabularies only in English were used for searches in the other databases.

Controlled and free vocabularies in the search for the studies were identified for the terms: *stroke* , *acute* , and *protocol* , which were combined through the use of Boolean operators AND and OR, which made it possible to obtain greater specificity in the literature review. The AND operator restricted the search, since it needed to contain all the searched terms, while the OR grouped the terms with the same meaning, expanding the search. Thus, the search strategies specifically for this search were as follows: ( *stroke* OR other synonyms) AND ( *acute* OR other synonyms) AND ( *protocol* OR other synonyms), which are presented in the Supplementary File .

The results of the searches after the bibliographic survey in the databases were exported to Rayyan QCRI online review application of the Qatar Computing Research Institute, 14 which enabled eliminating duplication and selecting publications by two independent reviewers according to the aforementioned criteria. The articles were initially selected by reading the title and abstract of the articles, and a third reviewer decided to include or exclude them when there was disagreement between the articles selected by the reviewers. Then, the full reading of the materials was performed, and as these were relevant to the review, data extraction was started using a specific instrument adapted from Ursi, 15 which included the following items: article title, journal name, authors, study location, language and year of publication, study objective, study type, study population/sample, data collection sources, comparison group, study variables, study duration, statistical treatment, and main results.

The results of the studies included in the present review were narratively synthesized and the methodological quality of the articles was evaluated through the use of instruments proposed by the The Joanna Briggs Institute. 13 In this case, we use the instrument that assesses cohort studies, and another that assesses cross-sectional studies, allowing to indicate the number of items adequately addressed in the studies according to the number of items provided by the instruments (11 items provided for cohort studies and 8 items for cross-sectional studies). It is noteworthy that no study was excluded due to the methodological quality assessment.

## RESULTS

**Figure 1:**
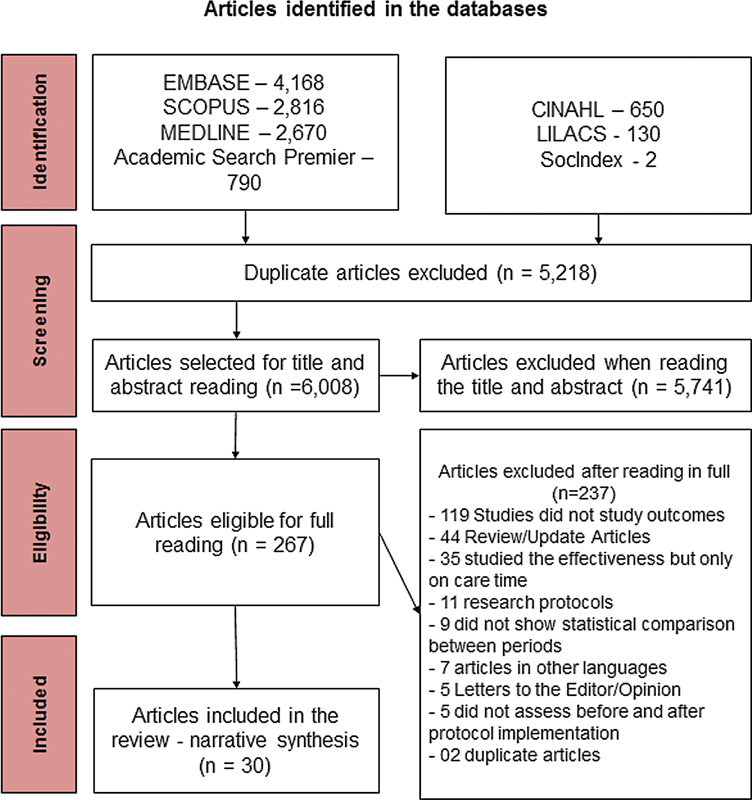
Selection flowchart of scientific publications included in the systematic review on the effect of implementing care protocols on the outcomes of acute ischemic stroke.

A total of 11,226 publications were retrieved in the databases using the above-mentioned descriptors, of which 5,218 were excluded due to duplication. Next, 5,741 were excluded after reading the titles and abstracts of 6,008 publications. Thus, 237 selected materials were considered eligible for full reading, of which 30 were included in the study ( Figure 1 ). The articles were published in the following years: 6 (20.0%) in 2019, 16 17 18 19 20 21 2 (6.7%) in 2018, 22 23 2 (6.7%) in 2017, 24 25 8 (26.7%) in 2016, 26 27 28 29 30 31 32 33 2 (6.7%) in 2015, 34 35 5 (16.7%) in 2014, 36 37 38 39 40 4 (13.3%) in 2012, 41 42 43 44 and 1 (3.3%) in 2011 45 ( Table 1 ).

**Table 1:** Description of the articles included in the systematic review of the literature on the effect of implementing care protocols on the outcomes of acute ischemic stroke

Authors / Journal / Year / Country	Study design*	Objective(s)	Population ( *n* )
Ye et al. 16 /Stroke Vasc Neurol/2019/China	Before-and-after cohort	Evaluate the effectiveness of the Shenzhen acute ischemic stroke emergency map to optimize access to thrombolysis	6,843 before and 8,268 after, of which 568 underwent thrombolysis before and 802 after
Yang et al. 17 /J Stroke Cerebrovasc Dis/2019/USA	Retrospective, interrupted, and observational time series	Assess the effectiveness of the nurse-directed stroke code in improving the recognition and diagnosis time of cases in the hospital	124 patients
Madhok et al. 18 /J Stroke Cerebrovasc Dis/2019/USA	Transversal retrospective	Evaluate whether the prehospital care protocol increases the thrombolysis percentage in a door-to-needle time of up to 45 minutes	112 before and 236 after, of which 50 underwent thrombolysis before and 45 after
Ajmi et al. 19 /BMJ Qual Saf/2019/Norway	Cohort	Describe the project to improve the quality of care for stroke, which aims to reduce the door-to-needle time and improve case outcomes	446 before and 204 after
de Belvis et al. 20 /Int J Health Care Qual Assur/2019/Italy	Pre-post retrospective observation	Investigate the effect of implementing a new clinical course in patients with acute ischemic stroke	483 before and after
Silsby et al. 21 /Intern Med J/2019/Australia	Retrospective	Assess whether changes to a protocol could improve the treatment time of acute ischemic stroke cases	143 before and 134 after, of which 30 received thrombolysis before and 14 after
Nguyen-Huynh et al. 22 /Stroke/2018/USA	Before-and-after cohort	Present the results of Kaiser Permanente Northern California's stroke protocol according to door-to-needle time, use of thrombolysis, and symptomatic intracranial hemorrhage rates	310 before and 557 after
Zakaria et al. 23 /Int J Stroke/2018/Egypt	Observational prospective	Investigate obstacles to implementing reperfusion therapy, identify the need for corrective actions and measure the impact of implementing specific measures to improve it	261 before and 284 after
Koge et al. 24 /J Neurol Sci/2017/Japan	Retrospective	Evaluate the efficacy and safety of the standardized protocol for in-hospital stroke	25 before and 30 after
Cheng et al. 25 /J Stroke/2017/China	Cohort	Evaluate the impact of the national HECAL-Stroke project on improving treatment with intravenous thrombolysis	149,921 patients
Zinkstok et al. 26 /PLoS One/2016/Netherlands	Before-and-after cohort	Reduce door-to-needle time to ≤ 30 minutes by optimizing in-hospital stroke treatment	373 patients
Liang et al. 27 /Australasian Physical and Engineering Sciences in Medicine / 2016 / China	Cohort	Determine whether application of lean principles for flow optimization could accelerate the onset of thrombolysis	13 before and 43 after
Li et al. 28 /Stroke/2016/China	Prospective	Assess the change in the care quality of stroke by comparing adherence to the measures recommended by the guidelines before and after implementing these initiatives	12,173 before and 19,604 after
Moran et al. 29 /J Stroke Cerebrovasc Dis/2016/USA	Retrospective cohort	Assess the impact of providing neurocritical nursing care, as covered by the “stroke code” on delays in treating patients who have received thrombolysis	44 before and 122 after
Hsieh et al. 30 /PLoS One/2016/Taiwan	Cohort	Demonstrate improved quality of acute ischemic stroke treatment through a collaborative learning model	13,181 patients
Ibrahim et al. 31 /J Stroke Cerebrovasc Dis/2016/Qatar	Cohort	Evaluate the effect of the acute thrombolysis protocol on the “door-to-needle time” and on the prognosis of acute stroke cases	102 before and 102 after
Rai et al. 32 /J Neurointerv Surg/2016/USA	Cohort	Present the results of a quality improvement process aimed at reducing stroke treatment time	64 before and 30 after
Mascitelli et al. 33 /J Neurointerv Surg/2016/USA	Retrospective	Assess the impact of evidence and a redesigned stroke protocol	32 before and 37 after
Kendall et al. 34 /Emerg Med J/2015/England	Cohort	Describe how the Stroke 90 project was set up and what interventions were implemented, report the results and discuss lessons learned from it.	136 before and 215 after
Atsumi et al. 35 /J Stroke Cerebrovasc Dis/2015/Japan	Cohort	Investigate whether prehospital and in-hospital thrombolysis indicators improved after using a municipal transport protocol	2,049 patients
Van Schaik et al. 36 /J Stroke Cerebrovasc Dis/2014/Netherlands	Cohort	Reduce the delay in in-hospital treatment of patients with acute ischemic stroke by implementing a standard operating procedure	41 before, 90 in the immediate intervention period and 185 in the late period
Chen et al. 37 /PLoS One/2014/China	Cohort	Investigate the impact of the stroke code on the performance of thrombolytic therapy and on the functional outcomes of patients	91 before and 2,016 after
Handschu et al. 38 /Int J Stroke/2014/Germany	Cohort	Implement and obtain certification of a quality management system in the telestroke network	2,049 before, 2,047 after immediate intervention and 2,324 in the late period
Fonarow et al. 39 /JAMA/2014/USA	Cohort	Analyze the temporal trend of the door-to-needle time for administering thrombolysis and if there was an improvement in the clinical results of stroke cases	27,319 before and 43,850 after
Ruff et al. 40 /Stroke/2014/USA	Retrospective	Evaluate whether incorporating a stroke protocol significantly modified the median image-door and needle-holder times	1,413 before and 925 after
Ford et al. 41 /Stroke/2012/USA	Cohort	Compare the efficiency and safety metrics of using a stroke care protocol before and after its implementation	132 before and 87 after
Lin et al. 42 /Circ Cardiovasc Qual Outcomes/2012/USA	Cohort	Evaluate the association of pre-notification of the emergency medical service with the results of treatment of acute ischemic stroke	122,791 without and 249,197 with protocol
Tai et al. 43 /Intern Med J/2012/Australia	Cohort	Perform an analysis of the stroke code to reduce the time-to-needle and image-holder and to increase the performance of thrombolysis	96 before and 189 after
O'Brien et al. 44 /J Clin Neurosci/2012/Australia	Pre- and postintervention prospective cohort	Determine whether introducing a prehospital notification scheme reduces the time for thrombolysis onset and increases the use of this treatment	67 without and 42 with protocol
Sung et al. 45 /Stroke Res Treat/2011/China	Cohort	Determine whether the protocol modification shortened the in-hospital delay and facilitated thrombolytic therapy	338 before and 139 after

Notes: *The study design was mentioned according to how it was mentioned in the original article; **The authors did not present the study population before or after implementing the protocol.

All 30 articles 16 17 18 19 20 21 22 23 24 25 26 27 28 29 30 31 32 33 34 35 36 37 38 39 40 41 42 43 44 45 included in the present review were published in English, and 10 (33.3%) were performed in the American continent, 17 18 22 29 32 33 39 40 41 42 6 (20%) in Europe, 19 20 26 34 36 38 10 (33.3%) in Asia, 16 24 25 27 28 30 31 35 37 45 3 (10%) in Oceania 21 43 44 and 1 (3.3%) in Africa/Asia. 23

From the included articles, 10 (33,3%) were performed in the United States, 17 18 22 29 32 33 39 40 41 42 6 (20%) in China, 16 25 27 28 37 45 3 (10%) in Australia, 21 43 44 2 (6.7%) in Japan, 24 35 2 (6.7%) in the Netherlands 26 36 and 7 (23.3%) articles (1 in each) of the following countries: Norway, Italy, Germany, Egypt, Taiwan, Qatar, and England 19 20 23 30 31 34 38 ( Table 1 ).

The objectives found in the scientific production regarding the systematic review on the outcomes of the use of protocols in the therapeutic approach to acute ischemic stroke are presented in Table 1 .

An increase in the reperfusion therapy rate was identified after implementing the protocol in 17 studies (70.8%) 16 22 23 25 26 27 30 31 33 34 36 37 38 39 40 43 44 of 24 16 17 22 23 25 26 27 28 29 30 31 32 33 34 35 36 37 38 39 40 42 43 44 45 which evaluated this aspect. Among 8 articles that evaluated the length of stay, 20 22 28 31 38 39 41 43 2 (25.0%) 28 31 studies identified a decrease. Regarding the postdischarge prognosis, 5 (45.5%) articles identified an improvement in this outcome 19 27 28 31 35 out of 13 19 24 26 27 28 31 33 35 37 38 41 43 45 that evaluated it. From the 19 studies 18 19 21 22 24 25 26 27 30 31 33 36 37 39 41 42 43 44 45 that addressed the symptomatic intracranial hemorrhage rate, 2 (10.5%) 30 39 identified a decrease in this rate. A decrease in mortality was mentioned in 3 (25%) articles 19 28 39 out of 12 19 20 26 27 28 29 30 33 37 38 39 43 that evaluated this outcome ( Table 2 ).

**Table 2:** Description of the results and evaluation of the methodological quality of the articles included in the systematic literature review on the effect of implementing care protocols on the outcomes of acute ischemic stroke

Authors EMQ	Thrombolysis rate	Thrombectomy rate	Length of stay	Modified Rankin Scale (mRS)	Symptomatic intracranial hemorrhage	Death
Ye et al. 16 8/11	Increased from 8.3 to 9.7% ( *p =* 0.003)	Increased from 0.9 to 1.6% (p < 0.001)	DNM	DNM	DNM	DNM
Yang et al. 17 5/11	13% before and 20% after ( *p =* 0.99)	6% before and 11% after ( *p =* 0.33)	DNM	DNM	DNM	DNM
Madhok et al. 18 4/8	DNM	DNM	DNM	DNM	0% before and 2% after ( *p =* 0.34)	DNM
Ajmi et al. 19 8/11	DNM	DNM	DNM	mRS 5–6 after 90 days reduced from 12.2 to 3.5% ( *p =* 0.021)	1.5% before and 0.5% after ( *p =* 0.306)	After 90 days it reduced from 9.1 to 3.5% ( *p =* 0.049)
de Belvis et al. 20 6/11	DNM	DNM	Increased from M 44.7 to 65.1 days ( *p* < 0.001)	DNM	DNM	After 30 days, 8.1% before and 9.7% after ( *p =* 0.52)
Silsby et al. 21 5/11	DNM	DNM	DNM	DNM	23.3% before and 14.3% after ( *p =* 0.48)	DNM
Nguyen-Huynh et al. 22 8/11	Increased from 13.1 to 17.6% (p < 0.001)	DNM	Mdn 3.5 days before and 3.1 after ( *p =* 0.14)	DNM	2.2% before and 3.8% after ( *p =* 0.21)	DNM
Zakaria et al. 23 8/11	Increased from 11.3 to 81.1% a	Increased from 1.9 to 13.5% a	DNM	DNM	DNM	DNM
	Reperfusion increased from 2.7 to 12.3% ( *p* < 0.05)						
Koge et al. 24 5/11	DNM	DNM	DNM	mRS ≤ 2 at discharge 28.0% before and 33.3% after ( *p =* 0.67)	8.0% before and 3.3% after ( *p =* 0.45)	DNM
Cheng et al. 25 5/11	Increased from 3.0 to 4.5% (p < 0.05)	DNM	DNM	DNM	The overall rate was 8.6% for all years of study ( *p* > 0.05)	DNM
Zinkstok et al. 26 7/11	The annual number of thrombolysis events increased from 17 (Mdn) to 55a	DNM	DNM	mRS 0–2 after 90 days 38.9% in the intervention period and 52.3% after a	3.0% before and 4.4% after ( *p* < 0.156)	After 90 days 17.9% before and 18.2% after
Liang et al. 27 8/11	Increased from 37.1 to 64.5% ( *p =* 0.026)	DNM	DNM	mRS 0–2 after 90 days increased from 30.7 to 75% ( *p =* 0.012)	0 before, 4.4% in the 1 ^st^ period and 0 in the 2 ^nd^ period ( *p =* 0.482)	7.7 before, 4.4% in the 1st period and 0 in the 2nd period ( *p =* 0.491)
Li et al. 28 8/11	14.1% before and 18.3% after	DNM	Reduced from Mdn 14 (IQR 11-20) days to 13 (IQR 9-16) (p < 0.001)	mRS ≤2 increased from 67.3% to 75.0% (p < 0.001)	DNM	Reduced from 4,1 to 1,1% ( *p* < 0.001)
Moran et al. 29 5/11	( *p* > 0.05)	32% before and 21% after ( *p =* 0.16)	DNM	DNM	DNM	18% before and 12% after ( *p =* 0.33)
Hsieh et al. 30 5/11	Increased from 1.2 to 4.6% ( *p* < 0.001)	DNM	DNM	DNM	Reduced from 11.0 to 5.6% (p < 0.001)	After 30 days 4.2% before and 4.1% after ( *p =* 0.914)
Ibrahim et al. 31 5/11	Increased from 4.0 to 11.8% ( *p* < 0.0001)	DNM	Reduced from Mdn 7 (IQR 4-13) days to 4 (IQR 2-6) days ( *p* < 0.001)	mRS 0–2 after 90 days increased from 47.1% to 73.3% ( *p* < 0.001)	5.9% before and 5.9% after ( *p =* 0.99)	7.8% before and 3.9% after ( *p =* 0.23)
Rai et al. 32 8/11	40.6% before and 43.3% after ( *p =* 0.8)	DNM	DNM	DNM	DNM	DNM
Mascitelli et al. 33 6/11	53.1% before and 54.1% after ( *p =* 0.938)	Increased from 2.9 cases per month to 7.4 ( *p =* 0.04)	DNM	mRS 0-2 at discharge, 21.9% before and 18.9% after ( *p =* 0.7740)	9.4% before and 10.8% after ( *p =* 0.957)	15.6% before and 10.8% after ( *p =* 0.553)
Kendall et al. 34 8/11	Similar between periods ( *p =* 0.60)b	DNM	DNM	DNM	DNM	DNM
Atsumi et al. 35 8/11	Increased from 11.8% to 23.7% ( *p =* 0.0135)	DNM	DNM	mRS <2 after 30 days 23.5% before 34.8% after ( *p =* 0.045)	DNM	DNM
Van Schaik et al. 36 8/11	51% before and 66% after a	DNM	DNM	DNM	7.3% before and 4.9% after ( *p =* 0.606)	DNM
Chen et al. 37 7/11	Increased from 5.0% to 19.5% ( *p* < 0.001)	DNM	DNM	mRS ≤ 2 after 90 days 44.0% before and 50.5% after ( *p =* 0.298)	7.7% before and 4.6% after ( *p =* 0.285)	6.6% before and 3.2% after ( *p =* 0.216)
Handschu et al. 38 7/11	Increased from 2.6 to 8.6% ( *p* < 0.001)	DNM	M 7.9 days before and 7.9 after a	mRS 4–6 23.4% before and 23.3% after a	DNM	5.8% before and 4.6% after a
Fonarow et al. 39 7/11	Increased from 5.7 to 8.1% ( *p* < 0.001)	DNM	Mdn 5 (IQR 3-8) before and 5 (IQR 3-7) after a	DNM	Decreased from 5.7 to 4.7% (p < 0,001)	After the intervention, in-hospital mortality was less likely to occur (adjusted OR, 0.89 [95%CI: 0.83–0.94]) ( *p* < 0.001)
Ruff et al. 40 6/11	Increased from 8.2 to 15.4% (p < 0.001)	DNM	DNM	DNM	DNM	DNM
Ford et al. 41 7/11	DNM	DNM	Mdn 4 (IQR 3-7) days before and 3 (IQR 2-6) after ( *p =* 0.056)	mRS ≤ 2 after 90 days 49% before and 43% after ( *p =* 0.34)	3.0% before and 3.4% after ( *p =* 1.0)	DNM
Lin et al. 42 5/11	64.0% before and 73.0% after a	DNM	DNM	DNM	6.0% before and 5.8% after ( *p* = 0.4020)	DNM
Tai et al. 43 5/11	Increased from 9.0% before to 17.3% after a	DNM	M 13 (SD 19) days before and M 11 (SD 17) after (p = 0.348)	mRS < 2 at discharge 68% before and 74% after ( *p =* 0.303)	5% before and 7% after ( *p =* 0.483)	13% before and 13% after ( *p =* 0.863)
O'Brien et al. 44 8/11	Increased from 7 to 19% ( *p =* 0.03)	DNM	DNM	DNM	There was one case of symptomatic intracranial hemorrhage during both periods	DNM
Sung et al. 45 7/11	11.8% before and 15.1% after ( *p =* 0.331)	DNM	DNM	mRS 0–1 35% before and 28.6% after ( *p =* 0.611)	12.5% before and 9.5% after ( *p =* 1.000)	DNM

Abbreviations: CI, confidence interval; DNM, did not mention; EMQ, Evaluation of Methodological Quality; IQR, interquartile range; M, mean; Mdn, median; OR, odds ratio; SD, standard deviation.

Notes: ^a^ Did not present p-value; ^b^ Did not present percentage.

The questions of the methodological quality assessment instruments contained questions that were not applicable to the studies, such as identifying and managing confounding variables and implementing strategies to minimize follow-up losses, reducing the number of well-evaluated items in all articles by three ( Table 2 and Supplementary File ). Thus, 11 studies included all the items considered by The Joanna Briggs Institute as indispensable for the studies carried out. 16 19 22 23 27 28 32 34 35 36 44 The main limitations found in the articles comprise unclear information about the study population 17 21 24 25 26 29 30 31 37 38 39 41 43 45 and a possible information bias in collecting exposure and outcome measures in studies that used secondary sources. 17 18 20 21 24 25 29 30 31 33 40 42 43

## DISCUSSION

Most of the articles evaluated in the present systematic review were carried out in countries with a high level of economic and social development. Formal schooling rates in these countries with a high quality of life standard are higher and there is significant public and private investment in research and incentives to publicize achievements in neurological care. In addition, it is important to highlight the composition of health services in developed countries that provide care for stroke cases and require training of the entire care network for diagnosing suspicion of cases, as well as a reorganization of the care flow in such a way as to lead affected individuals to specialized services and with adequate infrastructure for their treatment, which includes hiring specialized teams, the presence of neuroimaging technologies and availability of medications to perform chemical or mechanical thrombolysis and cranial surgeries.

Optimized emergency department and prehospital systems such as stroke response teams, ambulance prenotification, and direct transport from screening to neuroimaging exams are essential to maximize the benefit of reperfusion therapies, which are heavily time-dependent. 46 Thus, the increase in reperfusion rates occurs when there is availability and integrity of protocols, training and infrastructure in prehospital care associated with an introduction of complete hospital protocols involving all relevant professionals. 47

An increase in the reperfusion therapy rate was identified in 17 studies (70.8%) after implementing the protocol. Of these, 16 (94.1%) articles found an increase in the thrombolysis rate 16 22 23 25 26 27 30 31 34 36 37 38 39 40 43 44 and 3 (17.6%) reported an increase in thrombectomy, 16 23 33 assuming that such a positive outcome is a result of all the impacts arising from implementing stroke protocols that provide efficient screening and reorganization of pre- and intrahospital care for instituting timely treatment, especially with activation of the prehospital stroke code and implementing telemedicine, which takes the extension of thrombolysis to small and medium-sized hospitals 48 and provides expert guidance for more complex treatment decisions in distant areas. 46

Despite these results, three studies, 28 29 45 which showed no significant difference in reperfusion rates with the implementation of the protocol, indicated difficulties in diagnosing the complexity degree of the stroke and also a possible low adherence of the teams to the changes as weaknesses for an increase in reperfusion rates. In addition, lack of knowledge about the symptoms of the disease and emergency treatment can prevent people and their families from seeking immediate care, 49 thus hindering the performance of reperfusion therapies.

Only two studies 28 31 identified a decrease in the length of hospital stay, meaning that it seems that the recovery time of cases after treatment does not depend on a reduced time of prehospital care. However, it is worth emphasizing the need for further studies in relation to this perspective in order to clarify what affects the length of hospital stay.

The impact of the implanted protocols on the prognosis of the patient after discharge was remarkable in almost half of the evaluated articles. Such a prognosis is identified as “good” when the results of the modified ranking scale is ≤ two (on a scale of zero to six). The improvement in the prognosis after discharge depends on the time between stroke onset, the call for help, and establishment of the treatment itself, so that it is essential to raise awareness of lay people to recognize the signs and symptoms of stroke, in addition to prioritizing patient care after suspected diagnosis and establishing a sequence of actions filed between all care sectors in order to make treatment possible in a timely manner. As this response time is improved, more patients will be able to benefit from the thrombus elimination procedures and consequently reduce the sequelae resulting from the stroke and restore their health.

Among other complications of thrombolysis, 19 (46.66%) studies 18 19 21 22 24 25 26 27 30 31 33 36 37 39 41 42 43 44 45 addressed the symptomatic intracranial hemorrhage rate, with only 2 (10.5%) 30 39 identifying a decrease in this rate. In view of this, it is worth emphasizing the need to develop treatments or establish safer therapeutic dosages that have an impact on reducing the rate of symptomatic intracranial hemorrhage, given the low effectiveness of implementing protocols in this outcome.

The decrease in mortality was cited in 25.0% of the articles 19 28 39 that evaluated this outcome. The decrease in the mortality rate involves preparing the team for quick decision-making and conducting care of cases, constituting aspects identified when comparing hospitals whose protocol was implemented with hospitals that did not implement it. 45 Among the possible obstacles to improving the mortality rate are the lack of a qualified team, few physicians familiar with the types of treatment, lack of coagulation tests, lack of standardized protocol in the unit, and absence of a hemodynamic team. 20 27 29 33

The time factor is crucial in the care of acute stroke, and the delay can cause irreversible damage to the patient, which is reflected in lethality. Thus, implementing a stroke protocol sometimes is part of a quality improvement intervention 50 and requires reorganizing the health system and readjusting the transport network to direct stroke cases to accredited and qualified hospitals, in addition to implementing screening processes with training professionals for care, rapid assessment and referral of cases and adequacy of the flow of patients in the stroke care network. All of this is necessary in providing quality care for acute stroke, as one of the great challenges for instituting reperfusion treatment in stroke ischemic conditions is the response time of the health system in such an emergency situation. In addition, it is noteworthy that state or regionalized acute stroke treatment systems are increasingly being promoted and developed with the objective of integrating general hospital units and comprehensive stroke centers, 51 as they are essential in developing countries and in small towns whose care network does not offer specialized care to the affected cases.

No study was excluded from the present review in assessing the methodological quality; however, there is a need for many studies to better elucidate the population studied in order to show the similarities between the groups studied. The limitation found about possible information bias is overcome by > 50% of the studies that performed prospective data collection.

The present study was limited by the impossibility of relating the outcomes of using protocols in stroke care with their composition and characteristics, since they were not always described in detail in the studies. In addition, gray literature that could contribute to the study of outcomes of implementing the use of protocols in the care of stroke cases was not included, and it was not possible to perform a meta-analysis or evaluate the quality of evidence in the present systematic review.

In conclusion, we identified the importance of implementing protocols in the care of acute ischemic stroke cases regarding increased performance of reperfusion therapies, such as thrombolysis and thrombectomy, and a good functional outcome with improved prognosis after discharge. However, it is necessary to emphasize the need for treatments or adequacy of therapeutic dosages that focus on reducing the length of hospital stay and the occurrence of symptomatic intracranial hemorrhage and that impact case survival with a reduction in mortality.

The use of well-defined pre- and intrahospital protocols can modify the outcomes of acute ischemic stroke cases, with specific attributions defined for each care level and that mobilize and integrate the various health services in the care network. To this end, it is essential to establish public policies aimed at increasing the capacity to respond to and manage stroke cases by developing actions aimed at health education of lay people and professionals for recognizing the signs and symptoms of a suspected case and for timely decision-making, as well as for the sustainability of using protocols in healthcare service routines.
