# Supplementary material for: Effect of implementing care protocols on acute ischemic stroke outcomes: a systematic review
Source: Arq Neuropsiquiatr. 2023 Mar 22;81(2):173–85. doi: 10.1055/s-0042-1759578 (PMC10033200; doi:10.1055/s-0042-1759578)
Supplement: Supplementary file 1 — Supplementary Material [file 10-1055-s-0042-1759578-s210322.pdf]

Table 1. Bibliographic search strategies used in the systematic review on the effectiveness of the urgent care protocols for acute ischemic stroke outcomes, according to the researched databases, Ribeirão Preto, São Paulo, Brazil, 2020.

Database	Search strategy
Embase®	(stroke:ti,ab,kw OR 'cerebrovascular accident':ti,ab,kw OR 'cerebrovascular accidents':ti,ab,kw OR cva:ti,ab,kw OR cvas:ti,ab,kw OR ischemia:ti,ab,kw OR 'cerebral vascular accident':ti,ab,kw OR 'cerebral vascular accidents':ti,ab,kw OR 'cerebral vascular insufficiency':ti,ab,kw OR 'cerebro vascular accident':ti,ab,kw OR 'cerebro vascular accidents':ti,ab,kw OR 'cerebrovascular injury':ti,ab,kw OR 'cerebrovascular insufficiency':ti,ab,kw OR 'ischaemic cerebral attack':ti,ab,kw OR 'ischemic cerebral attack':ti,ab,kw OR 'brain ischemia':ti,ab,kw OR 'brain ischemias':ti,ab,kw OR 'cerebral ischemia':ti,ab,kw OR 'cerebral ischemias':ti,ab,kw OR 'ischemic encephalopathies':ti,ab,kw OR 'ischemic cerebral infarct':ti,ab,kw OR 'brain accident':ti,ab,kw OR 'brain attack':ti,ab,kw) AND (protocols:ti,ab,kw OR protocol:ti,ab,kw) AND (emergencies:ti,ab,kw OR emergency:ti,ab,kw OR urgencies:ti,ab,kw OR urgency:ti,ab,kw OR acute:ti,ab,kw)
Scopus	TITLE-ABS-KEY(Stroke OR "Cerebrovascular Accident" OR "Cerebrovascular Accidents" OR CVA OR CVAs OR Ischemia OR "Cerebral vascular accident" OR "Cerebral vascular accidents" OR "Cerebral vascular insufficiency" OR "Cerebro vascular accident" OR "Cerebro vascular accidents" OR "Cerebrovascular injury" OR "Cerebrovascular insufficiency" OR "Ischaemic cerebral attack" OR "Ischemic cerebral attack" OR "Brain Ischemia" OR "Brain Ischemias" OR "Cerebral Ischemia" OR "Cerebral Ischemias" OR "Ischemic Encephalopathies" OR "Ischemic cerebral infarct" OR "Brain accident" OR "Brain attack") AND TITLE-ABS-KEY(Protocols OR Protocol) AND TITLE-ABS-KEY(Emergencies OR Emergency OR Urgencies OR Urgency OR Acute)
MEDLINE	((Stroke OR "Cerebrovascular Accident"(Title/Abstract) OR "Cerebrovascular Accidents"(Title/Abstract) OR CVA OR CVAs OR Ischemia OR "Cerebral vascular accident"(Title/Abstract) OR "Cerebral vascular accidents"(Title/Abstract) OR "Cerebral vascular insufficiency"(Title/Abstract) OR "Cerebro vascular accident"(Title/Abstract) OR "Cerebro vascular accidents"(Title/Abstract) OR "Cerebrovascular injury"(Title/Abstract) OR "Cerebrovascular insufficiency"(Title/Abstract) OR "Ischaemic cerebral attack"(Title/Abstract) OR "Ischemic cerebral attack"(Title/Abstract) OR "Brain Ischemia"(Title/Abstract) OR "Brain Ischemias"(Title/Abstract) OR "Cerebral Ischemia"(Title/Abstract) OR "Cerebral Ischemias"(Title/Abstract) OR "Ischemic Encephalopathies"(Title/Abstract) OR "Ischemic cerebral infarct"(Title/Abstract) OR "Brain accident"(Title/Abstract) OR "Brain attack"(Title/Abstract)) AND (Protocols(Title/Abstract) OR Protocol(Title/Abstract))) AND (Emergencies(Title/Abstract) OR Emergency(Title/Abstract) OR Urgencies(Title/Abstract) OR Urgency(Title/Abstract) OR Acute(Title/Abstract))
LILACS[a]	(Stroke OR "Cerebrovascular Accident" OR "Cerebrovascular Accidents" OR CVA OR CVAs OR Ischemia OR "Cerebral vascular accident" OR "Cerebral vascular accidents" OR "Cerebral vascular insufficiency" OR

	"Cerebro vascular accident" OR "Cerebro vascular accidents" OR "Cerebrovascular injury" OR "Cerebrovascular insufficiency" OR "Ischaemic cerebral attack" OR "Ischemic cerebral attack" OR "Brain Ischemia" OR "Brain Ischemias" OR "Cerebral Ischemia" OR "Cerebral Ischemias" OR "Ischemic Encephalopathies" OR "Ischemic cerebral infarct" OR "Brain accident" OR "Brain attack" OR "Acidente Vascular Cerebral" OR "Acidente Cerebral Vascular" OR "Acidente Cerebrovascular" OR AVC OR "Acidente Vascular do Cérebro" OR "Acidente Vascular Encefálico" OR "Acidentes Cerebrais Vasculares" OR "Acidentes Cerebrovasculares" OR "Acidentes Vasculares Cerebrais" OR Apoplexia OR "AVC Agudo" OR AVE OR "Derrame Cerebral" OR "Accidente Cerebrovascular" OR "Accidente Cerebral Vascular" OR "Accidente Vascular Cerebral" OR "Accidente Vascular Encefálico" OR "Accidente Vascular del Cerebro" OR "Accidentes Cerebrovasculares" OR Apoplejía OR "Ataque Cerebral" OR "Isquemia Encefálica" OR "Encefalopatía Isquémica" OR "Isquemia Cerebral" OR "Cerebral Infarction" OR "Cerebral Infarctions" OR “Infarto Cerebral “ OR "Infarto do Cerebro" OR "Icto cerebral" OR "Ictus cerebral") AND (Protocols OR Protocol OR Protocolos OR Protocolo) AND (Emergencies OR Emergency OR Urgencies OR Urgency OR Acute OR Emergências OR Emergência OR Urgências OR Urgência OR Agudo OR Aguda OR "Urgencias Médicas" OR Emergencias OR Emergencia OR Urgencias OR Urgencia)
Academic Search Premier; CINAHL; SocINDEX	AB (Stroke OR "Cerebrovascular Accident" OR "Cerebrovascular Accidents" OR CVA OR CVAs OR Ischemia OR "Cerebral vascular accident" OR "Cerebral vascular accidents" OR "Cerebral vascular insufficiency" OR "Cerebro vascular accident" OR "Cerebro vascular accidents" OR "Cerebrovascular injury" OR "Cerebrovascular insufficiency" OR "Ischaemic cerebral attack" OR "Ischemic cerebral attack" OR "Brain Ischemia" OR "Brain Ischemias" OR "Cerebral Ischemia" OR "Cerebral Ischemias" OR "Ischemic Encephalopathies" OR "Ischemic cerebral infarct" OR "Brain accident" OR "Brain attack") AND AB (Protocols OR Protocol) AND AB (Emergencies OR Emergency OR Urgencies OR Urgency OR Acute)

[a] Words of the title, abstract and subject were searched in the Regional Portal of the Virtual Health Library, and the LILACS filter was used in consulted databases.
The filter 2011 to 2020 was applied for the publication period in all databases.

Table 2. Methodological quality evaluation of articles included in the systematic review on the effectiveness of the urgent care protocols for acute ischemic stroke in reducing care times, according to the researched databases, Ribeirão Preto, São Paulo, Brazil, 2020.

	1. Were the two groups similar and recruited from the same population?	2. Were the exposures measured similarly to assign people to both exposed and unexposed groups?	3. Was the exposure measured in a valid and reliable way?	4. Were confounding factors identified?	5. Were strategies to deal with confounding factors stated?	6. Were the groups/participants free of the outcome at the start of the study (or at the moment of exposure)?	7. Were the outcomes measured in a valid and reliable way?	8. Was the follow up time reported and sufficient to be long enough for outcomes to occur?	9. Was follow up complete, and if not, were the reasons to loss to follow up described and explored?	10. Were strategies to address incomplete follow up utilized?	11. Was appropriate statistical analysis used?
Ye et al.[16]	Y	Y	Y	NA	NA	Y	Y	Y	Y	NA	Y
Yang et al.[17]	U	Y	N	NA	NA	Y	N	Y	Y	NA	Y
Madhok et al.[18]	Y	Y	N	Y	NA	NA	N	Y	-	-	-
Ajmi et al.[19]	Y	Y	Y	NA	NA	Y	Y	Y	Y	NA	Y
de Belvis et al.[20]	Y	Y	N	NA	NA	Y	N	Y	Y	NA	Y
Silsby et al.[21]	U	Y	N	NA	NA	Y	N	Y	Y	NA	Y
Nguyen-Huynh et al.[22]	Y	Y	Y	NA	NA	Y	Y	Y	Y	NA	Y
Zakaria et al.[23]	Y	Y	Y	NA	NA	Y	Y	Y	Y	NA	Y
Koge et al.[24]	U	Y	N	NA	NA	Y	N	Y	Y	NA	Y
Cheng et al.[25]	U	Y	N	NA	NA	Y	N	Y	Y	NA	Y
Zinkstok et al.[26]	U	Y	Y	NA	NA	Y	Y	Y	Y	NA	Y
Liang et al.[27]	Y	Y	Y	NA	NA	Y	Y	Y	Y	NA	Y
Li et al.[28]	Y	Y	Y	NA	NA	Y	Y	Y	Y	NA	Y
Moran et al.[29]	U	Y	N	NA	NA	Y	N	Y	Y	NA	Y
Hsieh et al.[30]	U	Y	N	NA	NA	Y	N	Y	Y	NA	Y
Ibrahim et al.[31]	U	Y	N	NA	NA	Y	N	Y	Y	NA	Y
Rai et al.[32]	Y	Y	Y	NA	NA	Y	Y	Y	Y	NA	Y
Mascitelli et al.[33]	Y	Y	N	NA	NA	Y	N	Y	Y	NA	Y
Kendall et al.[34]	Y	Y	Y	NA	NA	Y	Y	Y	Y	NA	Y
Atsumi et al.[35]	Y	Y	Y	NA	NA	Y	Y	Y	Y	NA	Y
Van Schaik et al.[36]	Y	Y	Y	NA	NA	Y	Y	Y	Y	NA	Y
Chen et al.[37]	U	Y	Y	NA	NA	Y	Y	Y	Y	NA	Y

Handschu et al.[38]	U	Y	Y	NA	NA	Y	Y	Y	Y	NA	Y
Fonarow et al.[39]	U	Y	Y	NA	NA	Y	Y	Y	Y	NA	Y
Ruff et al.[40]	Y	Y	N	NA	NA	Y	N	Y	Y	NA	Y
Ford et al.[41]	U	Y	Y	NA	NA	Y	Y	Y	Y	NA	Y
Lin et al.[42]	U	Y	N	NA	NA	Y	N	Y	Y	NA	Y
Tai et al.[43]	U	Y	N	NA	NA	Y	N	Y	Y	NA	Y
O'Brien et al.[44]	Y	Y	Y	NA	NA	Y	Y	Y	Y	NA	Y
Sung et al.[45]	U	Y	Y	NA	NA	Y	Y	Y	Y	NA	Y

Legend: N - No; U – unclear; Y – Yes; NA= not applicable

*Questions for cross-sectional studies are different from the cohort studies: 1. Were the criteria for inclusion in the sample clearly defined?; 2. Were the study subjects and the setting described in detail?; 3. Was the exposure measured in a valid and reliable way?; 4. Were objective, standard criteria used for measurement of the condition?; 5. Were confounding factors identified?; 6. Were strategies to deal with confounding factors stated?; 7. Were the outcomes measured in a valid and reliable way?; 8. Was appropriate statistical analysis used?
